# SdiA, a Quorum-Sensing Regulator, Suppresses Fimbriae Expression, Biofilm Formation, and Quorum-Sensing Signaling Molecules Production in *Klebsiella pneumoniae*

**DOI:** 10.3389/fmicb.2021.597735

**Published:** 2021-06-21

**Authors:** Thaisy Pacheco, Ana Érika Inácio Gomes, Nathália Maria Gonçalves Siqueira, Lucas Assoni, Michelle Darrieux, Henrietta Venter, Lúcio Fábio Caldas Ferraz

**Affiliations:** ^1^Laboratório de Biologia Molecular de Microrganismos, Universidade São Francisco, Bragança Paulista, Brazil; ^2^Health and Biomedical Innovation, Clinical and Health Sciences, University of South Australia, Adelaide, SA, Australia

**Keywords:** *Klebsiella pneumonia*, SdiA regulator, cell division, quorum sensing, type 1 fimbriae, biofilm

## Abstract

*Klebsiella pneumoniae* is a Gram-negative pathogen that has become a worldwide concern due to the emergence of multidrug-resistant isolates responsible for various invasive infectious diseases. Biofilm formation constitutes a major virulence factor for *K. pneumoniae* and relies on the expression of fimbrial adhesins and aggregation of bacterial cells on biotic or abiotic surfaces in a coordinated manner. During biofilm aggregation, bacterial cells communicate with each other through inter- or intra-species interactions mediated by signallng molecules, called autoinducers, in a mechanism known as quorum sensing (QS). In most Gram-negative bacteria, intra-species communication typically involves the LuxI/LuxR system: LuxI synthase produces *N*-acyl homoserine lactones (AHLs) as autoinducers and the LuxR transcription factor is their cognate receptor. However, *K. pneumoniae* does not produce AHL but encodes SdiA, an orphan LuxR-type receptor that responds to exogenous AHL molecules produced by other bacterial species. While SdiA regulates several cellular processes and the expression of virulence factors in many pathogens, the role of this regulator in *K. pneumoniae* remains unknown. In this study, we describe the characterization of *sdiA* mutant strain of *K. pneumoniae*. The *sdiA* mutant strain has increased biofilm formation, which correlates with the increased expression of type 1 fimbriae, thus revealing a repressive role of SdiA in fimbriae expression and bacterial cell adherence and aggregation. On the other hand, SdiA acts as a transcriptional activator of cell division machinery assembly in the septum, since cells lacking SdiA regulator exhibited a filamentary shape rather than the typical rod shape. We also show that *K. pneumoniae* cells lacking SdiA regulator present constant production of QS autoinducers at maximum levels, suggesting a putative role for SdiA in the regulation of AI-2 production. Taken together, our results demonstrate that SdiA regulates cell division and the expression of virulence factors such as fimbriae expression, biofilm formation, and production of QS autoinducers in *K. pneumoniae*.

## Introduction

*Klebsiella pneumoniae* is a Gram-negative bacterium responsible for various diseases that has become a worldwide concern due to the increase in cases of severe infections in the community (Keynan and Rubinstein, 2007; Lin et al., 2010; Holt et al., 2015). Much of *K. pneumoniae* pathogenicity comes from its ability to form biofilms (Li et al., 2014; Paczosa and Mecsas, 2016), which are microbial communities that grow attached to surfaces and typically surrounded by a matrix of self-produced extracellular polymeric substances (Hall-Stoodley et al., 2004). Biofilm formation by *K. pneumoniae*, which can occur on biotic and abiotic surfaces, represents a relevant mechanism to protect the bacterium from host immunity and antimicrobial agents (Li et al., 2014; Paczosa and Mecsas, 2016).

During biofilm aggregation, the bacterial cells communicate with each other through inter- or intra-species interactions mediated by a mechanism called quorum sensing (QS). By this process, bacteria produce and detect specific signaling molecules to coordinate gene expression according to the bacterial cell density (Bassler et al., 1993; Miller and Bassler, 2001). Signaling is mediated by chemical molecules, known as autoinducers (AI), that determine two main types of cell-cell communication: AI-1 and AI-2 QS regulatory systems.

AI-2 QS system allows intra- and inter-species communication and utilizes cyclic furanones compounds as AI-2 signaling molecules. AI-2 is synthesized by LuxS synthase, a key enzyme of the activated methyl cycle that converts S-ribosylhomocysteine to homocysteine and 4,5-dihydroxy-2,3-pentanedione, which spontaneously rearranges to form the AI-2 molecules (Schauder et al., 2001; Kendall and Sperandio, 2014). Highly conserved *luxS* gene homologues have been found in several Gram-negative and Gram-positive bacteria (Bassler et al., 1993; Miller and Bassler, 2001), including *K. pneumoniae* (Balestrino et al., 2005). The regulatory network for AI-2 metabolism in *K. pneumoniae* also relies on *lsr* (comprized of *lsrACDBFG*) and *lsrRK* operons, which are located adjacent to one another in the genome but are transcribed divergently (Xavier and Bassler, 2005b; Pereira et al., 2009; Pereira et al., 2013). The first four genes of the *lsr* operon (*lsrACDB*) encode an ATP-binding cassette (ABC) transporter system that uptakes the AI-2 molecules, while the remaining genes, *lsrFG*, are required for processing AI-2 molecules following internalization. Once inside the cell, AI-2 is phosphorylated by the cytoplasmic kinase LsrK, and the activated phospho-AI-2 molecule binds to the transcriptional repressor LsrR and inactivates it. In the absence of phospho-AI-2, LsrR represses the transcription of the *lsr* operon and regulates its own expression by repressing the *lsrRK* operon. While LsrR represses the expression of both *lsrR* and *lsr* operon, cyclic adenosine monophosphate (cAMP) complexed with the cAMP-receptor protein activates their expression (Wang et al., 2005). In *K. pneumoniae*, the operons *lsr* and *lsrRK* are up-regulated in mature biofilm (Guilhen et al., 2016). Besides, previous studies with *K. pneumoniae luxS* and *lsrCD* mutant strains revealed a regulatory role of AI-2 QS system on biofilm formation and lipopolysaccharide synthesis (Ng and Bassler, 2009; Tavio et al., 2010).

AI-1 QS system utilizes *N*-acyl-L-homoserine lactones (AHLs) as autoinducers and represent the major QS system used by Gram-negative bacteria for intra-species communication and to monitor their own population density (Engebrecht and Silverman, 1984; Ng and Bassler, 2009). In most bacteria, the AI-1 QS mediated by AHLs involves a typical system composed of LuxI and LuxR proteins: LuxI is the enzyme that synthesizes AHLs molecules and LuxR is the cognate receptor that acts as a transcriptional regulator in response to the binding of the AHL autoinducers (Engebrecht and Silverman, 1984; Ng and Bassler, 2009). A variety of AHLs molecules have been identified, each differing in length, oxidation state at β-position, and saturation degree of the *N*-acyl side chain. Intriguingly, some Gram-negative bacteria encode LuxR receptors but do not produce AHLs because they lack LuxI synthase. These LuxR-type receptors without their corresponding LuxI synthase are known as “solo” or “orphan” receptors (Fuqua, 2006; Patankar and González, 2009). For instance, bacteria from the genera *Salmonella*, *Escherichia*, and *Klebsiella* harbor no *luxI* gene homologues in their genome and, therefore, they are considered non-AHL producers (Michael et al., 2001). Nonetheless, these enteropathogens encode SdiA, an orphan LuxR-type receptor that senses and responds to AHLs synthesized by other species of bacteria (Ahmer, 2004; Janssens et al., 2007). Although most members of the *Enterobacteriaceae* family contains solo *sdiA*, species from the genus *Pantoea* and *Erwinia* harbor *luxI* homologs which represent descendants of the ancient LuxI protein paired with SdiA (Sabag-Daigle and Ahmer, 2012).

SdiA stands for “suppressor of cell division inhibition” and directly regulates gene expression by binding to regulatory elements, termed SdiA-box, located at the promoter region of the target genes (Yamamoto et al., 2001). Reports indicate that the nucleotide sequence of the SdiA-box consists of the sequence AAAA (with minor variations) at both ends, intercalated with a spacer sequence that can vary from 8, 10, to 18 nucleotides (Yamamoto et al., 2001; Shimada et al., 2014; Lu et al., 2017; Ma et al., 2020). SdiA regulates the transcription of the target genes by complexing with AHLs synthesized by other bacterial species (Michael et al., 2001; Smith and Ahmer, 2003; Ahmer, 2004), in response to synthetic AHLs (Shimada et al., 2014; Styles et al., 2020), or even in the absence of AHLs (Yamamoto et al., 2001; Dyszel et al., 2010; Shimada et al., 2014; Nguyen et al., 2015). Besides, non-AHL molecules have been identified as SdiA ligands, such as xylose (Yao et al., 2007) and the endogenous ligand 1-octanoyl-*rac*-glycerol (Nguyen et al., 2015). Indole has also been suggested to influence SdiA-mediated gene transcription (Lee et al., 2007; Lee et al., 2009), although this claim is contraditory, since some authors have reported that the effects of indole on *Escherichia coli* and *Salmonella enterica* is not mediated by *sdiA* (Sabag-Daigle et al., 2012; Kohli et al., 2018).

Acting as a transcriptional regulator, SdiA has been implicated in the regulation of several cellular processes including cell division (Sitnikov et al., 1996) and in the expression of virulence factors such as antibiotic resistance, motility and biofilm formation (Kanamaru et al., 2000; Sharma et al., 2010; Antunes et al., 2010; Tavio et al., 2010; Culler et al., 2018; Ma et al., 2020). Bacterial cell division relies on the *ftsQAZ* operon, which encodes the FtsQ, FtsA, and FtsZ proteins responsible for recruiting and assembling cell division machinery in the septum. Regulation of this operon is complex and involves multiple promoters and several transcriptional regulators (Joseleau-Petit et al., 1999). In *E. coli*, two promoters located upstream of *ftsQ* gene are responsible for independent transcriptional regulation of the full operon: P1 promoter is controlled by the stationary-phase Sigma factor RpoS, while P2 promoter is controlled by SdiA (Wang et al., 1991; Sitnikov et al., 1996). SdiA plays an important role during cell division because it acts as a positive regulator in the expression of *ftsQAZ* operon (Wang et al., 1991), and *in vitro* assays confirmed DNA-binding activity of purified SdiA to the *ftsQ* promoter (Shimada et al., 2014). Regarding virulence factors, SdiA regulates biofilm formation through regulation of fimbriae and curli genes (Culler et al., 2018). Studies have shown that fimbriae play an important role during biofilm formation by *K. pneumoniae* (Schroll et al., 2010; Alcántar-Curiel et al., 2013), but the role of SdiA in this process has not yet been addressed in this bacterium.

*K. pneumoniae* encodes several types of fimbriae. The most studied fimbriae are those of type 1, type 3 and the common pilus encoded by the *fim*, *mrk*, and *ecp* gene clusters, respectively (Alcántar-Curiel et al., 2013; Li et al., 2014). Type 3 fimbriae mediate adhesion to several types of cells and they are essential for biofilm formation on abiotic surfaces, thus playing a role in the development of infections in catheterized patients (Struve et al., 2009; Stahlhut et al., 2012; Li et al., 2014). On the other hand, type 1 fimbriae are essential for urinary tract infection (Struve et al., 2008). Due to the high affinity for mannose residues present on the bladder cells surface (Rozen and Skaletsky, 2000), type 1 fimbriae promote adhesion and invasion of epithelial bladder cells, leading to the formation of biofilm-like intracellular bacterial communities (Rosen et al., 2008). The expression of the *fim* cluster is regulated by a mechanism known as phase variation (van der Woude and Baumler, 2004). The phase variation involves the *fimS* element, a DNA fragment located immediately upstream to the *fimA* gene that harbors the promoter region of *fim* cluster and has the capacity to suffer inversion of its orientation. Thus, depending on the orientation of *fimS*, the expression of *fim* gene cluster is activated or inactivated and the bacterium can shift from a fimbriated (phase ON) to a non-fimbriated (phase OFF) phenotype. Two recombinases, encoded by the *fimE* and *fimB* genes located upstream to *fimS*, control the inversion of the *fimS* element (van der Woude and Baumler, 2004).

While the role of SdiA in regulating the expression of several virulence factors in many pathogens is well documented, little is known about the role of this regulator in *K. pneumoniae*. Therefore, the present work aimed to investigate the role of SdiA in *Klebsiella pneumoniae* pathogenicity by assessing biofilm formation, fimbriae expression and production of quorum sensing autoinducers on an *sdiA* mutant strain of *K. pneumoniae*.

## Materials and Methods

### Bacterial Strains and Culture Conditions

*Klebsiella pneumoniae* strain ATCC 10031 and an isogenic mutant strain deficient for *sdiA* gene were used throughout this study. Bacterial strains were routinely grown in Lysogeny Broth (LB; BD, United States) at 37°C with shaking at 200 rpm, and on LB agar under static cultures. Bacterial growth was monitored by measuring the optical density (O.D.) of the cultures at wavelength of 600 nm (O.D._60__0n__m_) using the GeneQuant Spectrophotometer (GE Healthcare). Antibiotics were added when appropriated at the following concentrations: ampicillin at 100 μg/mL, kanamycin at 25 μg/mL, chloramphenicol at 25 μg/mL, and erythromycin at 50 μg/mL.

For Reverse Transcription Quantitative Real-Time PCR (RT-qPCR) analyses and detection of autoinducers type 2, the strains were grown in LB medium with the addition of 2% glucose, since glucose stimulates *K. pneumoniae* to produce more AI-2 (Zhu et al., 2012).

To investigate the phenotypic effects of AHL on the *K. pneumoniae* strains, the assays carried out in this study were conducted using bacterial cells cultured in the absence or in the presence of 2 μM (final concentration) of the synthetic AHL *N*-Octanoyl-L-homoserine lactone (C8-HSL, Sigma-Aldrich). Previous studies have indicated that C8-HSL is an effective AHL autoinducer for both *E. coli* and *Salmonella enterica* SdiA (Michael et al., 2001; Yao et al., 2006; Lee et al., 2007; Kim et al., 2013; Shimada et al., 2014).

For indirect measurements of AI-2 molecules produced by the *K. pneumoniae* strains we used the reporter strain *Vibrio campbellii* MM32 (ATCC^®^ BAA-1121*^TM^*). This strain is unable to produce AI-2 and to sense AHL due to mutations on *luxS* and *luxN* receptor genes, respectively. Autoinducer Bioassay medium (AB medium) was used to culture *V. campbellii* and also as the assay medium for AI-2 detection (Bassler et al., 1993), and consisted of 0.3 M NaCl, 0.05 M MgSO_4_ and 0.2% vitamin-free acid casamino, adjusted to pH 7.5 with KOH and sterilized by autoclaving. After reaching room temperature, 10 mL of 1 M potassium phosphate (pH 7.0), 10 mL of 0.1 M L-arginine and 20 mL of glycerol were added for each liter of the initial solution.

### Generation of *K. pneumoniae sdiA* Mutant Strain

SdiA-deficient ATCC 10031 mutant strain was generated using TargeTron Gene Knockout System (Sigma-Aldrich), following a protocol previously standardized by us (Gomes et al., 2021). The TargeTron system produces an RNA-protein complex (RNP) that inserts a modified group II intron of *Lactococcus lactis* (L1. LtrB Intron) permanently on the coding region of the target gene. The knockout renders the mutant strain resistant to kanamycin antibiotic, because the group II intron RNA harbors a kanamycin resistance gene (*kan*^*R*^). A computer algorithm at Sigma-Aldrich TargeTron Design website was used to identify the most efficient target site on *sdiA* gene (Supplementary Table 1). The website also provided the nucleotide sequence of three primers (Supplementary Table 2) used to mutate (re-target) the intron by PCR reactions. The mutated 350 bp PCR fragment was cloned into the pACD4K-C vector provided by the manufacturer. Subsequently, the recombinant pACDK-4-C vector were transformed into *E. coli* DH5α strain to obtain clones. The pACD4K-C vector contains a T7 promoter to express the intron and RNP, and a source of T7 RNA Polymerase was provided by plasmid pAR1219. Therefore, competent ATCC 10031 cells were cotransformed with pAR1219 and recombinant pACDK-4-C, and incubated in LB broth containing ampicillin and chloramphenicol at 37°C for 18 h with shaking at 200 rpm. Next, a new incubation of ATCC 10031 cells was performed with fresh LB broth under the same conditions. When the O.D._60__0n__m_ reached 0.2, the expression of RNP was induced with the addition of 0.1 M IPTG and incubation at 30°C for 30 min with shaking at 200 rpm. Then, the cells were centrifuged for 2 min at 10,000 *g*, resuspended in fresh LB broth, and incubated again at 30°C for 1 h. Colonies grown on agar plate supplemented with kanamycin were selected after 18 h of incubation at 37°C. Since gene knockout by TargeTron System is based on the insertion of *kan*^*R*^ gene inside *sdiA*, the mutant strain of *K. pneumoniae* ATCC 10031 was renamed *sdiA*::*kan*^*R*^.

The complemented strain *sdiA*::*kan*^*R*^_comp_ was obtained by introducing the *sdiA* gene back into the mutant strain. For this, a DNA fragment comprising the entire coding region of *sdiA* plus the 3′ and 5′ flanking regions was PCR amplified using primers listed on Supplementary Table 3. The DNA fragment was inserted on pCR^TM^2.1 vector (Invitrogen^TM^) previously cloned with erythromycin-resistance gene. Chemically competent *sdiA*::*kan*^*R*^ was transformed with the recombinant vector and plated on LB agar supplemented with 50 μg/mL erythromycin. Complemented strains were recovery by screening erythromycin-resistant colonies.

### Growth Pattern and Optical Microscopy Analysis

The wild-type ATCC 10031, mutant *sdiA*::*kan*^*R*^, and complementary sdiA::*kan*^*R*^_comp_ strains were separately inoculated into LB medium and grown until saturation (overnight) at 37°C under shaking. The next day, the culture was diluted 1:100 in fresh LB and the bacterial growth was monitored every 15 min by measuring the O.D._60__0n__m_. Growth curves were constructed by plotting the O.D._60__0n__m_ values against time. To investigate the morphology of the bacterial cells, 10 μL of each culture were harvested at the indicated O.D._60__0n__m_, stained with fuchsine and visualized under optical microscopy. Results were recorded under 1000 × magnification. Three independent cultures of each *K. pneumoniae* strain were conducted for the growth pattern and the optical microscopy analysis.

### Biofilm Mass Assay and Pellicle Formation at the Air-Liquid Interface

Biofilm formation assays were carried out conducted in 96-well microtiter plates as described previously (Gomes et al., 2021). Saturated cultures of the bacterial strains were harvested by centrifugation and resuspended in LB broth to a final concentration of 10^6^ cells/mL. 150 μL of each cell suspension were applied in 96-well microtiter plates containing 150 μL of LB supplemented or not with C8-HSL. The plates were incubated at 37°C under static conditions for 8, 24, 48, and 72 h of incubation. After each time, the medium was discarded, and the biofilm mass was gently rinsed with PBS. The wells were left to dry for 5 min and then stained with 0,1% of crystal violet for 15 min at room temperature. After staining, the crystal violet was discarded, the wells were rinsed 3 times with PBS and left to dry for 5 min. The biofilm-associated crystal violet was solubilized with 200 μL acetic acid (30%, v/v), and the absorbance of the acetic acid containing the eluted dye was measured at O.D._60__0n__m_ with a Biotek Microplate reader. Biofilm formation assays were conducted in duplicates from three independent cultures of each *K. pneumoniae* strain.

We also investigated the formation of pellicle in air-liquid interface by the *K. pneumoniae* strains. This is a biofilm-like structure that requires a great organization due to the lack of solid surface for fixation. To investigate pellicle formation, the strains were inoculated overnight and subsequently grown in LB medium until O.D._60__0n__m_ of 0.6. The cultures were diluted to obtain a final density of 5 × 10^6^ CFU/mL. Six milliliters of each cell suspension were applied in glass tubes and incubated for 72 h under static conditions at 37°C. The formation of pellicles at the air-liquid interface in the glass tubes was recorded using a digital camera. Pellicle formation assays were performed in triplicate for each *K. pneumoniae* strain, and they were not conducted in presence of the autoinducer C8-HSL.

### Phase Variation Assay of the *fimS* Element

The orientation of the *fimS* element, which contains the promoter region of the cluster *fim*, was investigated according to a phase variation assay previously described (Struve et al., 2008). *K. pneumoniae* strains were grown to O.D._60__0n__m_ of 0.6 in LB broth at 37°C with shaking. Cells were harvested and the DNA were extracted with the *Wizard*^®^ *Genomic DNA Purification Kit* (Promega). DNAs were then used to PCR amplify an 817 bp fragment containing the invertible *fimS* promoter element using primers CAS168 and CAS169 (Supplementary Table 3). The amplified fragments were cut with *Hin*fI restriction enzyme and the pattern of the digested products was determined on 2% agarose gels stained with ethidium bromide. The *Hin*fI restriction site is asymmetrically located on the *fimS* element, which results in different cleaved fragments depending on the orientation of the phase switch: a phase switched to the ON orientation results in fragments of 212 and 605 bp, whereas a phase switched to the OFF orientation results in fragments of 321 and 496 bp. The assays were performed from two independent cultures for each strain of *K. pneumoniae*.

### Agglutination Assay

Yeast agglutination assays were performed to investigate the expression of type 1 fimbriae by the *K. pneumoniae* strains. The assays were conducted on Kline concavity slides as described previously, with minor modifications (Gomes et al., 2021). Overnight cultures of the bacterial strains were inoculated (1:100) into fresh LB broth supplemented or not with C8-HSL and cultured until they reached O.D._60__0n__m_ of 0.6. Bacteria were then mixed with 5% (w/v) suspension of *Saccharomyces cerevisiae* cells (Sigma-Aldrich) prepared in PBS. The intensity of the agglutination was documented using a digital camera. The agglutination of the yeast cells is specifically mediated by type 1 fimbriae since these fimbriae have great affinity for mannose, a highly abundant residue on yeast cell-surface. Therefore, the assays were also performed in the presence of 5% D-(+)-Mannose (Sigma-Aldrich) to confirm if the agglutination was indeed mediated by the type 1 fimbriae. Assays were carried out in triplicate for each *K. pneumoniae* strain.

### Indirect Detection of AI-2

The production of AI-2 molecules by the *K. pneumoniae* strains was indirectly measured using the reporter strain *Vibrio campbellii* MM32, as previously reported (Zhu et al., 2008). Initially, the strains were cultured overnight in LB broth containing 2% glucose. On the following day, the cultures were inoculated (1:100) into LB broth supplemented or not with C8-HSL, and samples were collected at O.D._60__0n__m_ of 0.2, 0.4, 0.6, 0.8, and 1.0. Cell-free conditioned culture supernatant was obtained by centrifuging the cultures at 10,000 *g* and passing the supernatant through a Millipore membrane filter (pore size of 0.22 μm). *V. campbellii* MM32 was grown overnight at 30°C at 200 rpm in AB medium and then diluted 1:5000 in fresh AB medium. 180 μL of the MM32 diluted culture were distributed in 96-well microtiter plates, followed by the addition of 20 μL of the cell-free conditioned culture supernatant. The mixtures were incubated with shaking at 30°C, and the luminescence were measured every 15 h in the equipament GloMax^®^ 96 Microplate Luminometer (Promega, Madison, WI, United States). Assays were carried out at least in triplicate for each *K. pneumoniae* strain. The results are expressed as arbitrary luminescence units and were obtained by dividing the light values measured on the experimental samples by the light values of the sterile LB culture medium.

### RNA Extraction and Real-Time Quantitative PCR Analysis

*K. pneumoniae* cells were grown in LB broth at 37°C with shaking at 200 rpm until O.D._60__0n__m_ of 0.2 and 0.6. Cell pellets were obtained after centrifugation, and total bacterial RNA were extracted by using the TRIzol^TM^ Max*^TM^* Bacterial RNA Isolation and MICROB*Enrich*^TM^ kits (Invitrogen^TM^), following the manufacturer’s instructions. After treatment with DNAse, 1 μg of total RNA was reverse transcribed in cDNA using the High-Capacity cDNA Reverse Transcription kit (Applied Biosystems^TM^), according to the manufacturer’s recommendations. Synthesized cDNA was used in RT-qPCR analyses, using primers listed in Supplementary Table 3.

Primers were designed using *Primer3 version 0.4.0* web-program^1^ (Rozen and Skaletsky, 2000). Reactions were performed in triplicates on the Applied Biosystems^®^ 7300 Real-Time PCR System equipment (ThermoFisher Scientific) with the Platinum^TM^ SYBR^TM^ Green qPCR SuperMix-UDG kit (Invitrogen^TM^), following the manufacturer’s instructions. RT-qPCR results were normalized using *rho* as endogenous gene, which encodes the transcription termination factor Rho (Gomes et al., 2018). The relative expression levels of the genes were calculated using the 2^–ΔΔCt^ relative quantification method (Livak and Schmittgen, 2001). GraphPad Prism 7.0 (GraphPad Software, Inc) was used for the statistical analyses.

### Purification of *K. pneumoniae* SdiA Protein and Electrophoretic Mobility Shift Assays (EMSA)

The coding region of *sdiA* gene was amplified by PCR using *K. pneumoniae* genomic DNA as template and primers containing the restriction sites for *Nde*I and *Xho*I (Supplementary Table 3). After digestion with *Nde*I and *Xho*I, the amplicon was cloned into the expression vector pET28a(+) (Sigma-Aldrich) at the corresponding sites, and the resulting recombinant vector was transformed into *E. coli* BL21(DE3). The expression and purification of the histidine-tagged recombinant SdiA protein (His-SdiA) were conducted as described elsewhere (Gomes et al., 2018), with some modifications. Some reports have shown the expression of recombinant SdiA from culture medium supplemented with AHLs autoinducers (Yao et al., 2006; Abed et al., 2014). Since we aimed to conduct EMSA with the *K. pneumoniae* SdiA protein in its apo form (i.e., not complexed with autoinducers), we did not add AHLs in the culture medium, as has been done by others (Kanamaru et al., 2000; Yamamoto et al., 2001; Wu et al., 2008; Kim et al., 2013; Shimada et al., 2014; Nguyen et al., 2015; Lu et al., 2017). In brief, transformed BL21(DE3) was grown in 500 mL of LB medium at 37°C to an O.D._60__0n__m_ of 0.4. At this moment, isopropyl-β-D-thiogalactopyranoside (IPTG, Sigma-Aldrich) was added to a final concentration of 1 mM and the culture was incubated for 4 h. After incubation, in-culture bacterial cell lysis was promoted by adding CelLytic^TM^ Express 1 mL Tablets (Sigma-Aldrich), following the manufacturer’s instructions. The lysed cells were centrifuged at 16,000 *g* for 15 min to obtain a clarified supernatant. His-SdiA was purified from the clarified supernatant by affinity chromatography under native conditions using Ni-NTA Agarose matrix (Qiagen), following to the manufacturer’s protocol. Eluted fractions containing the recombinant SdiA were pooled and dialyzed overnight at 4°C on storage buffer (50 mM Tris-HCl pH 8.0, 2 mM DTT, 0.5 mM EDTA, and 10 % glycerol v/v). The concentration of the purified His-SdiA was determined by the Bradford method and the purity was verified by SDS-PAGE analysis.

EMSA was conducted using the purified His-SdiA protein and DNA probes containing the promoter region of the indicated genes. The probes were generated by PCR amplifications using the primers and conditions displayed on Supplementary Table 3. As a negative control, a 220 base pairs DNA fragment was obtained by PCR amplifying a recircularized pCR^TM^2.1 vector (Invitrogen^TM^) without insert using primers M13 (Supplementary Table 3). Reactions were performed using 2 or 10 ρmol of the recombinant His-SdiA protein, previously equilibrated for 15 min at 37°C in 40 μL of 1X binding buffer containing 10 mM Tris-HCl pH 8.0, 50 mM KCl, 1 mM DTT, 0.1 mM EDTA, 2.5 mM MgCl2, and 2 % glycerol. After pre-incubation, 50 ηg of the DNA probes were added and the reaction mixture were incubated for 30 min at 37°C. To investigate the effects of AHLs on the binding affinity of SdiA, EMSA were carried out in the absence and in the presence of 2 or 4 μM of C8-HSL. Samples were submitted to electrophoresis at either 2% (w/v) agarose gel at 80 volts for 60 min in 1X sodium borate buffer (5 mM) or native non-denaturing 6% bis-acrylamide gel at 80 volts for 2 h in 1X TBE buffer (89 mM Tris, 89 mM boric acid, and 2 mM EDTA). DNA probe-SdiA complexes formed were visualized under UV light after staining the gels with ethidium bromide solution (0.5 μg/mL). Images of the DNA bands were recorded with Molecular Imager^®^ Gel Doc^TM^ XR System (Biorad) using the Image LabTM Software version 5.0 (Biorad).

## Results

### *K. pneumoniae* Depleted of SdiA Presents Impaired Cell Division and Abnormal Cell Morphology

**FIGURE 1:**
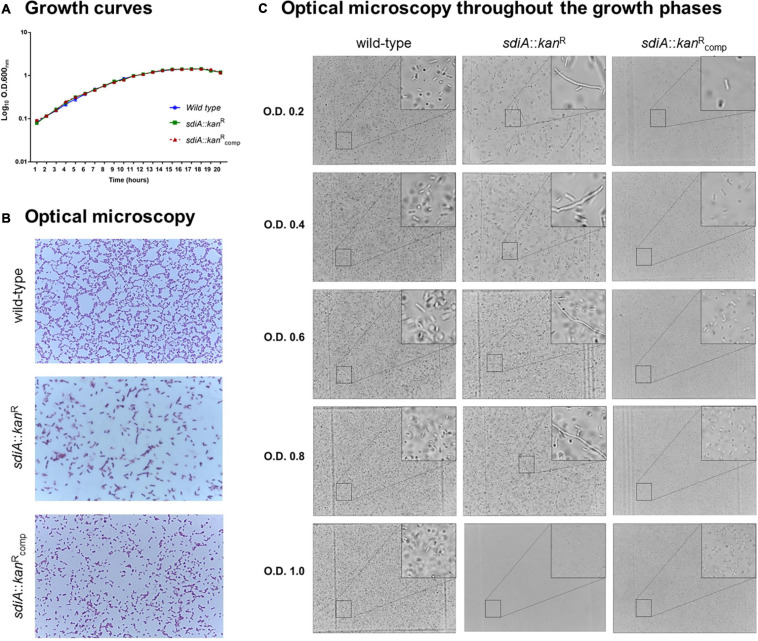
Lack of SdiA affects cell division and leads to morphological alterations of *K. pneumoniae* cells. **(A)** Growth curves of the wild-type, mutant (*sdiA*::*kan*^R^) and complemented (*sdiA*::*kan*^R^_comp_) strains are displayed as log scale of the optical density of the cultures of each strain measured at the indicated times. No differences in growth curves were observed among the strains. **(B,C)** Aliquots of the cultures of each strain were submitted to optical microscopy analyses to investigate the morphology of the bacterial cells. Disruption of *sdiA* led the mutant strain to exhibit a filamentous phenotype. The mutant strain recover the same morphology of the wild-type and complemented strains only at high cell densities (O.D._600 nm_ of 1.0).

In order to investigate the role of SdiA in *Klebsiella pneumoniae*, a knockout *sdiA* mutant strain was generated and phenotypically characterized. Firstly, growth curves of the wild-type (ATCC 10031), mutant *sdiA*::*kan*^R^ and complemented *sdiA*::*kan*^R^_comp_ strains were assessed. As shown in Figure 1A, no significant changes were observed in the growth curves of the mutant and the complemented strains in relation to the wild-type.

However, disruption of *sdiA* led the mutant strain to assume a filamentous phenotype (Figure 1B), denoting a failure in bacterial cell division. On the other hand, the morphology of the complemented strain was similar to the wild-type. Analyzing the morphology of the strains throughout growth stages (Figure 1C), we observed that the mutant strain presents the filamentous phenotype at O.D._60__0n__m_ of 0.2–0.8 and that the cell division of the mutant strain is recovered only at at high densities (O.D._60__0n__m_ of 1.0). No changes in the morphology of the wild-type and the complemented strain were observed throughout the growth stages (Figure 1C).

**FIGURE 2:**
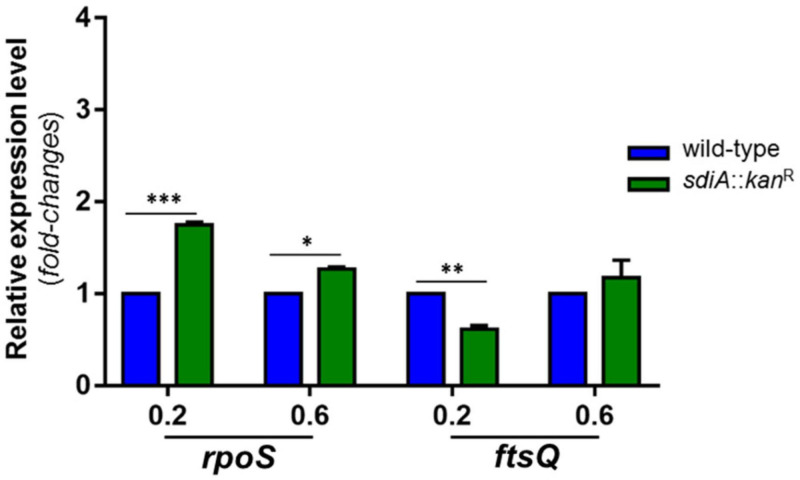
*K. pneumoniae* depleted of SdiA exhibits up-regulation of *rpoS* (stationary-phase Sigma factor RpoS) and down-regulation of *ftsQ* (gene cluster *fts* of cell division). The expression of *rpoS* and *ftsQ* genes was analyzed by RT-qPCR on the wild-type and mutant *K. pneumoniae* strains cultured at O.D._600 nm_ of 0.2 and 0.6. Error bars represent the standard deviation. Statistically significant differences between the strains were analyzed by multiple *t*-test (*p < 0.05; **p < 0.01; ***p < 0.001).

Since the mutant strain exhibited a filamentary shape in a manner dependent on the growth phase, we decided to investigate the expression pattern of *ftsQ*, from the *ftsQAZ* cell division gene cluster, and the *rpoS* gene, which encodes the stationary-phase Sigma factor RpoS. As displayed in Figure 2, the expression levels of *rpoS* in the mutant strain was almost twofold higher than the level of the wild-type strain at O.D._60__0n__m_ of 0.2, and slightly up-regulated at O.D._60__0n__m_ of 0.6. On the other hand, the expression levels of *ftsQ* in the mutant strain was slightly down-regulated at O.D._60__0n__m_ of 0.2 and unchanged at O.D._60__0n__m_ of 0.6, when compared to the wild-type strain.

### Lack of SdiA Increases Biofilm Formation and Yeast Cells Agglutination, and Leads to Down-Regulation of the Type 3 Fimbriae and Up-Regulation of Type 1 Fimbriae Expression

**FIGURE 3:**
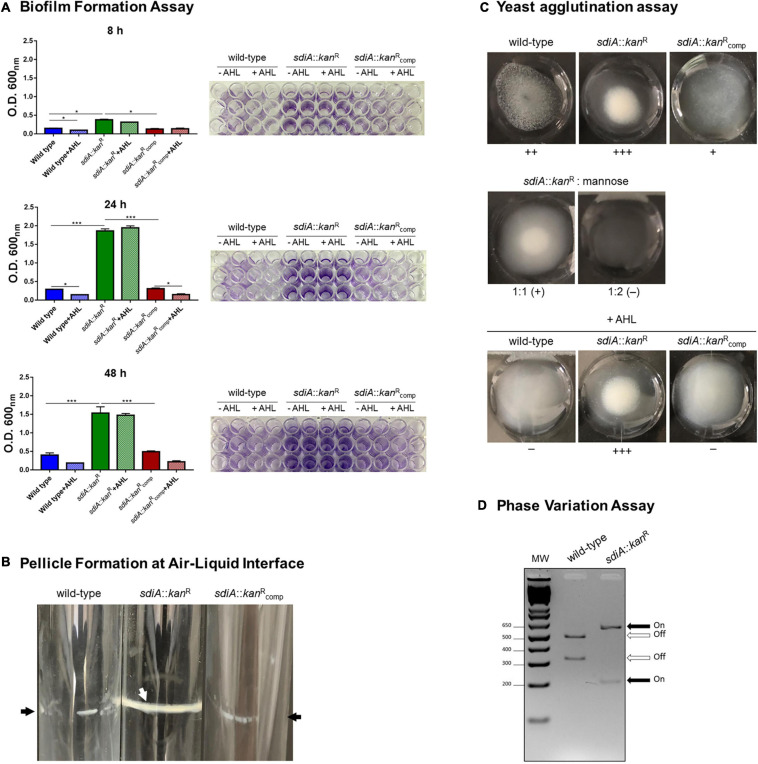
*K. pneumoniae* deficient in SdiA presents enhanced biofilm formation, yeast cells agglutination, and production of type 1 fimbriae. **(A)** The biofilm formation assays showed that the mutant strain (*sdiA*::*kan*^R^) forms more biofilm than the wild-type and complemented (*sdiA*::*kan*^R^_comp_) strains. The addition of the AHL C8-HSL reduced the biofilm formed by the wild-type and the complemented strains at the indicated incubation times but did not affect biofilm formation by the mutant strain. For this assay, the strains were incubated at 37 °C in 96-well microtiter plates under static condition for 8, 24, and 48 h, and then stained with crystal violet. The biofilm-associated crystal violet was solubilized and measured at O.D._600 nm_. Error bars represent the standard deviation. Statistically significant differences between the strains were analyzed using ANOVA (*p < 0.05; ***p < 0.001). The 96-well plates of each biofilm formation assay are shown to the right of the respective graphs. **(B)** The pellicle formation at the air-liquid interface assays showed that *K. pneumoniae* mutant strain presents thicker pellicle formation when compared to the wild-type and complemented strains. For this assay, strains were cultivated for 72 h at 37°C under shaking conditions. Black arrows indicate thin pellicle structure formation by the wild-type and complemented strain, while the white arrow indicates the formation of thick pellicle by the mutant strain. The images are from an individual representative experiment. **(C)** The yeast agglutination assays revealed that the mutant strain agglutinated yeast cells with more intensity than the wild-type strain. The absence of agglutination of yeast cells by the mutant strain in the presence of mannose confirms that the agglutination was mediated specifically by type 1 fimbriae. The addition of AHL reduced the agglutination by the wild-type and the complemented strains but had no effect on the agglutination of the yeast cells by the mutant strain. For yeast agglutination assays, cultures of the bacterial strains at O.D._600 nm_ of 0.6 were mixed with *Saccharomyces cerevisiae* cells, and the intensity of the agglutination was digitally documented. The images are from an individual representative experiment. **(D)** The phase variation assays showed the promoter region of the *fim* gene cluster at ON orientation in the mutant strain and at OFF orientation in the wild-type strain. For this assay, the *fimS* element, which contains the promoter region of the cluster *fim*, was PCR amplified using DNA extracted from the wild-type and mutant strains cultured in LB broth to O.D._600 nm_ of 0.6. The amplicons were cut with *Hin*fI and the digested fragments were resolved by electrophoresis on agarose gels. The *fimS* element at ON orientation results in fragments of 212 and 605 bp, whereas at OFF orientation results in fragments of 321 and 496 bp. The image is from an individual representative experiment.

The role of SdiA as a regulator of biofilm formation is well recognized in many pathogens, but it is still uncertain in *K. pneumoniae*. To assess whether SdiA is also involved in biofilm formation by *K. pneumoniae*, the ability of the *sdiA*::*kan*^R^ mutant strain to form biofilms was compared to the wild-type and complemented strains. As shown in Figure 3A, the biofilm formation by *sdiA*::*kan*^R^ was significantly superior than the wild-type after 8, 24, and 48 h of incubation, while the complemented *sdiA*::*kan*^R^_comp_ strain restored the pattern originally exhibited by the wild-type. The addition of the AHL C8-HSL had no effect on biofilm formation by the mutant strain, but reduced the biofilm formed by the wild-type and the complemented strains. Both wild-type and *sdiA*::*kan*^R^ strains were able to form a pellicle in the air-liquid interface (Figure 3B), but the mutant exhibited a thicker pellicle than the wild-type strain. Moreover, the phenotype was fully re-established on the complemented mutant strain.

Since fimbriae are considered as important mediators of bacterial adhesion and the loss of *sdiA* has resulted in more intense biofilm formation, we sought to investigate the production of fimbriae by the *K. pneumoniae* strains. Firstly, we compared the ability of the *K. pneumoniae* strains to agglutinate yeast cells. As displayed in Figure 3C, the mutant strain *sdiA*::*kan*^R^ was able to agglutinate yeast cells with more intensity than the wild-type, while the complemented strain partially recovered the phenotype exhibited by the wild-type. The addition of mannose abolished the agglutination of the yeast cells by the mutant strain, thus confirming that the agglutination was indeed mediated by type 1 fimbriae. The addition of AHL had no effect on the agglutination of the yeast cells by the mutant strain, but reduced the agglutination by the wild-type and the complemented strains.

Yeast agglutination is indicative of type 1 fimbriae expression because this type of fimbriae has a great affinity for mannose-containing receptors present on the surface of the yeast cells. To confirm that type 1 fimbriae expression is induced on the mutant strain, we sought to investigate the phase variation of the *fimS* invertible element containing the promoter region of the *fim* gene cluster. The phase variation assay indicated that the mutant strain presents the *fimS* element in the ON orientation (Figure 3D).

**FIGURE 4:**
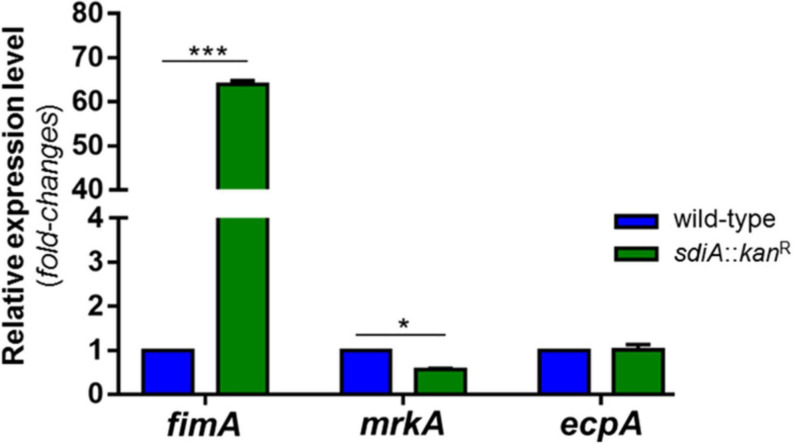
*K. pneumoniae* deprived of SdiA presents up-regulation of *fimA* (type 1 fimbriae) and down-regulation of *mrkA* (type 3 fimbriae). No change in the expression of *ecpA* (common pillus) was observed between the wild-type and mutant strains. The expression of the genes was analyzed by RT-qPCR on the wild-type and mutant *K. pneumoniae* strains cultured at O.D._60__0n__m_ of 0.6. Error bars represent the standard deviation. Statistically significant differences between the strains were analyzed by multiple *t*-test (*p < 0.05; ***p < 0.001).

To finally confirm the up-regulation of type 1 fimbriae in the mutant strain, analyses of fimbrial genes expression were conducted in the wild-type and *sdiA*::*kan*^R^ strains. As shown in Figure 4, the mutant strain exhibited significantly higher transcription levels of *fimA* (type 1 fimbriae) and slightly lower transcription of *mrkA* (type 3 fimbriae) than the wild-type strain. There was no difference statistically significant in the expression of *ecpA* (common pillus) between wild-type and mutant strains. These findings seem to indicate that the SdiA regulator modulates the expression of fimbriae in *K. pneumoniae*, by repressing the expression of type 1 fimbriae and inducing the expression of type 3 fimbriae.

### SdiA Deficient *K. pneumoniae* Strain Reaches Maximum Production of AI-2 Earlier

**FIGURE 5:**
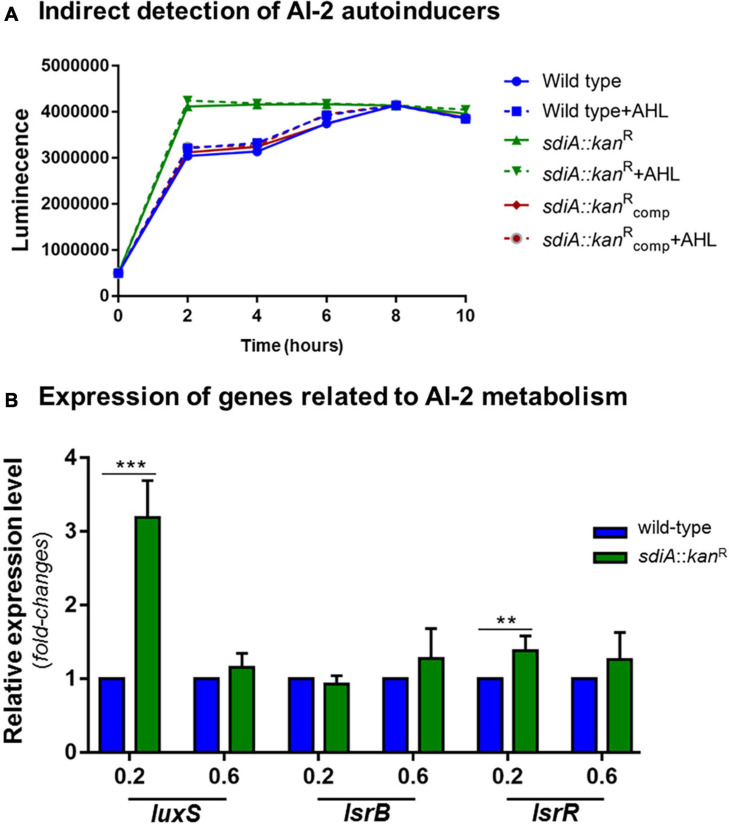
*K. pneumoniae* cells lacking SdiA regulator reach maximum production of AI-2 QS signaling molecules earlier and present up-regulation of *luxS* and *lrsR*. **(A)** Cell-free supernatants were collected from wild-type and *sdia*::*kan*^R^ mutant strains cultured at the indicated O.D. and tested for AI-2 production by indirect measuring the level of bioluminescence induced in the *V. campbellii* MM32 reporter strain. The mutant strain reaches maximum production of AI-2 after 2 h of culture, while the wild-type and complemented strains reached maximum production of AI-2 only after 8 h of culture. The addition of AHL had no effect on the production of AI-2 molecules by the *K. pneumoniae* strains. **(B)** The absence of SdiA causes up-regulation of *luxS* and *lsrR* genes, which encode the AI-2 synthase and the LsrR repressor, respectively. No change in the expression of *lsrB* (receptor of AI-2 molecules) was observed between the wild-type and mutant strains. Gene expression analysis were performed by RT-qPCR on the wild-type and mutant *K. pneumoniae* strains cultured at O.D._600 nm_ of 0.2 and 0.6. Error bars represent the standard deviation. Statistically significant differences between the strains were analyzed by multiple *t*-test (**p < 0.01; ***p < 0.001).

SdiA has been related to inter-species communication since this regulator senses and responds to AHLs synthesized by other bacteria species. To elucidate a possible relationship of SdiA with the inter-species communication mediated by the Autoinducers-2 (AI-2) QS signaling system, we used an indirect method based on the reporter strain *V. campbellii* to measure and compare the production of signal molecules AI-2 by the wild-type and the *sdiA* deficient mutant strains. According to Figure 5A, the mutant strain reaches maximum production of AI-2 after 2 h of culture, which corresponds to the lag phase of growth (O.D._60__0n__m_ of 0.1) according to the growth curve of the strain (Figure 1A). On the other hand, the wild-type and the complemented strains took longer and reached the same level of AI-2 as the mutant strain only after 8 h of culture, which corresponds to the mid-log phase of growth (O.D._60__0n__m_ of 0.6, Figure 1A). The addition of AHL had no apparent effect on the production of AI-2 molecules by the *K. pneumoniae* strains.

Based on these results, we proceeded to gene expression analyses of *luxS*, *lsrB*, and *lsrR*, genes related to AI-2 synthesis, uptake, and uptake regulation, respectively. As displayed in Figure 5B, the expression levels of *luxS* in the mutant strain was more than threefold higher than the wild-type strain at O.D._60__0n__m_ of 0.2 and slightly induced at O.D._60__0n__m_ of 0.6. No statistically significant difference was observed for the expression of *lsrB* between wild-type and mutant strains, whereas the expression levels of *lsrR* was slightly up-regulated at O.D._60__0n__m_ of 0.2 and unchanged at O.D._60__0n__m_ of 0.6 in the mutant strain, compared to that in the wild-type.

### SdiA Binds to the Promoter Region of *ftsQ*, *fimA*, *luxS, and lsrR*-*lsrA*

**FIGURE 6:**
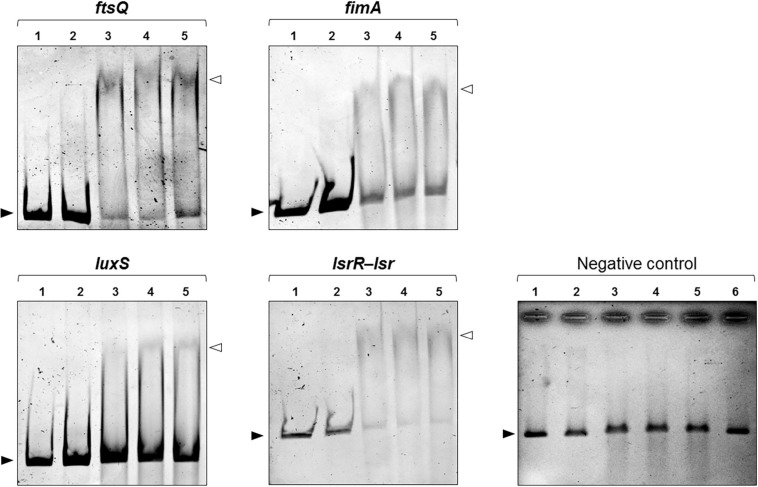
DNA Electrophoretic Mobility Shift Assays (EMSA) revealed that SdiA binds to the promoter region of *ftsQ*, *fimA*, and the intergenic region between the *lsrR* and *lsr* operon. For EMSA, 50 ηg of the DNA probes (in all lanes) were incubated with 2 ρmoles (lane 2) or 10 ρmoles (lanes 3–5) of the recombinant His-SdiA protein for 30 min at 37°C. To investigate the effects of AHL on the binding affinity of SdiA, 2 μM (lane 4) or 4 μM (lane 5) of C8-HSL were added to the binding reaction. Mobility shifts (indicated by open arrowheads) are observed only at higher concentration of purified SdiA (10 ρmoles, lanes 3–5). These shifts represent the formation of SdiA-DNA probe complexes and confirm the binding activity of SdiA on the DNA probes. The addition of C8-HSL have no apparent effect on the shifting mobility of the bands (lanes 4 and 5 compared). Closed arrowheads indicate the free DNA probes. No shifted bands were observed when SdiA was incubated with the negative control DNA probe. Lane 6 represents the incubation of the negative DNA probe (50 ηg) with 4 μM of C8-HSL in the presence of 10 ρmoles of the purified SdiA.

The significant up-regulation of *fimA* and *luxS* in the mutant strain led us to examine whether SdiA exerts a direct role on the expression modulation of these genes by binding on their promoter region. Firstly, bioinformatic analyses were employed to identify putative SdiA-boxes on the promoter region of *fimA*, *luxS* and on the intergenic region between *lsrR* and *lsrA*. The promoter region of the *ftsQAZ* operon was included in the analyses as a positive control because it is well known by previously published studies that SdiA binds in the promoter region of the operon and controls the transcription of *ftsQAZ* genes (Wang et al., 1991; Sitnikov et al., 1996; Yamamoto et al., 2001; Shimada et al., 2014). SdiA-binding sequences that resembles to the consensus sequence of SdiA-box (5′-AAAA(N_5__–__30_)AAAA-3′) were found on the promoter region of *fimA*, *luxS*, *lsrR*-*lsrA* and *ftsQAZ* (Supplementary Table 4). Next, EMSA was performed using the recombinant His-SdiA protein from *K. pneumoniae* and DNA fragments containing the putative SdiA-boxes as probes. As shown in Figure 6, shifted bands corresponding to SdiA-DNA probe complexes were observed only with 10 ρmol of the recombinant His-SdiA protein. The addition of C8-HSL, both 2 and 4 μM, had no apparent effect on the shifting mobility of the bands. No shifted bands were observed when His-SdiA was incubated with the negative control probe. These results indicate the direct binding of *K. pneumoniae* SdiA to the promoter region of *fimA*, *luxS*, *lsrR*-*lsrA* and *ftsQAZ*, and that this binding occurs in an AHL-independent manner.

## Discussion

In many pathogens, the SdiA regulator modulates the expression of several virulence factors, such as adherence and motility (Kanamaru et al., 2000; Sharma et al., 2010), multidrug resistance (Tavio et al., 2010), biofilm formation (Culler et al., 2018) and acid tolerance (Ma et al., 2020). Concerning *K. pneumoniae*, little is known about the role of SdiA in the pathogenesis of this bacterium and, to date, no member of the SdiA regulon had been described in *K. pneumoniae*. In the present study, we provided new insights into the role of SdiA in the expression of virulence factors by *K. pneumoniae* through characterization of a strain depleted from *sdiA* gene. We also investigated the presence of putative SdiA binding sites within the promoter region of genes responsible for the synthesis of type 1 fimbriae, bacterial cell division, and the metabolism of type 2 autoinducers.

First, we compared the growth pattern of the *sdiA* mutant strain with the wild-type and the complemented strains. Although no changes in bacterial growth were observed among the strains, *K. pneumoniae* cells lacking SdiA regulator presented a filamentary shape rather than the typical rod shape, revealing a failure in cell division by the mutant cells. Bacterial cell division relies on the *ftsQAZ* operon, which encodes essential cell division proteins. Regulation of this operon is complex and involves multiple promoters and several transcriptional regulators (Joseleau-Petit et al., 1999). Two promoter regions located upstream of *ftsQAZ* drive the transcription of the entire operon: P1 promoter is controlled by the stationary-phase Sigma factor RpoS and P2 promoter by SdiA (Joseleau-Petit et al., 1999). In *E. coli*, SdiA plays an important role in cell division because positively regulates the transcription of the *ftsQAZ* operon (Wang et al., 1991; Sitnikov et al., 1996; Shimada et al., 2014). In our study, *K. pneumoniae* cells depleted of the SdiA regulator presented down-regulation of the *ftsQ* gene, and EMSA analyses confirmed DNA-binding activity of the purified *K. pneumoniae* SdiA to the *ftsQAZ* promoter. These results suggest that the filamentary shape of the mutant strains seems to be due to the down-regulation of the *ftsQAZ* operon with consequent failure in septum division, and that SdiA acts as a transcriptional activator of the operon also in *K. pneumoniae*. We also observed a growth-phase dependent effect of SdiA, since the rod-shaped pattern was restored only at the stationary phase of growth. Since *ftsQAZ* expression mediated by SdiA shows cell density dependence and that Sigma Factor *RpoS* and SdiA act in a coordinated manner to guarantee *ftsQAZ* expression (Sitnikov et al., 1996), the restored rod-shaped pattern observed at stationary phase of growth may be attributed to RpoS. Although RpoS is responsible for gene expression activation when cells enter the stationary phase, the up-regulation of *rpoS* observed at the mutant strain throughout the phases of growth seems to compensate for the absence of *sdiA*.

Subsequently, we investigated how the lack of SdiA influences biofilm formation by *K. pneumoniae*. We observed greater biofilm and pellicle formation at air-liquid interface by the *sdiA* mutant strain as compared to the wild-type strain. Biofilm formation in the presence of exogenous AHL rendered distinct results: while no effect was observed for the mutant strain, the wild-type formed less biofilm. These results are in agreement with studies that reported an increase in biofilm formation and pellicle formation by *E. coli sdiA* mutant strains (Lee et al., 2007; Sharma et al., 2010; Culler et al., 2018). Similarly to our findings, these authors also noticed no change in biofilm formation by the mutant strain in the presence of AHL, whereas the wild-type strain has the formation of biofilm inhibited. Our results indicate that SdiA responds to AHL and represses biofilm formation in *K. pneumoniae*.

Expression of fimbriae is the first essential step in biofilm formation by *K. pneumoniae* and previous reports by others indicate that SdiA exerts its effects on biofilm formation by regulating the expression of fimbrial genes (Culler et al., 2018). Recently, Shimada and colleagues reported a direct link between SdiA regulator and expression of type 1 fimbriae encoded by *fim* gene cluster (Shimada et al., 2014). They showed that SdiA binds the promoter region of *fim* cluster, although the precise binding site has not been determined. These authors (Shimada et al., 2014) and others (Janssens et al., 2007) also described a decrease in *fimA* transcription on the wild-type *E. coli* strain in the presence of AHL. Although the current study has not analyzed gene expression in the presence of AHL, we observed that cells lacking SdiA were able to agglutinate yeast cells with greater intensity than the wild-type strain, and that this agglutination is due specifically to the production of type 1 fimbriae. Likewise observed in biofilm formation assays, no effect on yeast agglutination by the mutant strain was observed when exogenous AHL was added, while the wild-type had its ability to agglutinate yeast cells reduced. RT-qPCR analyses showed up-regulation of *fimA* – the gene encoding the major structural subunit of type 1 fimbriae – in the mutant strain. Corroborating this result, phase variation analyses revealed that the *fimS* element, which contains the promoter region of the type 1 *fim* gene cluster, is oriented at ON position in the mutant strain. Furthermore, EMSA analyses confirmed direct binding of SdiA to an SdiA-box located in the immediate vicinity of the *fimA* initiation codon. All results together suggest that SdiA has a repressive role in the expression of type 1 fimbriae in *K. pneumoniae*, and that the lack of this regulator resulted in a hyperfimbriated phenotype that rendered the mutant strain with greater ability to form biofilm and to agglutinate yeast cells. Thus far, a regulatory role of the QS system on attachment and biofilm maturation by *K. pneumoniae* had been described before, but involving type 2 QS signaling molecules (Balestrino et al., 2005; De Araujo et al., 2010). Here we show that SdiA, the LuxR-type receptor of the AI-1 QS system, has a suppressive role in bacterial adherence and biofilm aggregation by *K. pneumoniae* as well.

It has been reported that AHL autoinducers function as a folding switch for the regulation of some LuxR homologues (Zhu and Winans, 2001; Schuster et al., 2004; Urbanowski et al., 2004; Lee et al., 2006). In these cases, the receptors only assume their active functional structure and with higher DNA-binding activity when complexed with their cognate AHLs ligands. For these LuxR receptors, AHLs are required for proper folding of the protein, stabilizing it, and preventing it from degradation; *in vitro* purification of these LuxR receptors in the active soluble form requires the presence of AHL in the culture medium used for bacterial expression. Interestingly, AHL molecules elicited phenotypic changes in the wild-type *K. pneumoniae* strain, but they had no apparent effect on the DNA-binding affinity of the recombinant SdiA protein, as revealed by EMSA analyses. Moreover, the ability of the purified SdiA to bind to the promoter region of the target genes indicates that the recombinant protein is correctly folded, even though it was expressed in a culture medium without supplementation with AHLs. These results suggest that AHL may not be a folding switch for *K. pneumoniae* SdiA, and the proper folding of SdiA in the absence of AHL during *in vitro* expression may be due to the binding of a yet unknown endogenous non-AHL ligands such as 1-octanoyl-rac-glycerol (Nguyen et al., 2015), in a mechanism that has already been suggested for other bacterial species (Sabag-Daigle et al., 2015).

In this study, we observed DNA-binding activity of *K. pneumoniae* SdiA in an AHL-independent manner. Although previous reports have shown the higher affinity of SdiA with DNA when complexed with AHLs (Nguyen et al., 2015), our results are in agreement with other reports that show DNA-binding activity of SdiA even in the absence of ligands (Yamamoto et al., 2001; Dyszel et al., 2010; Shimada et al., 2014; Nguyen et al., 2015). For instance, Kim et al. (2013) reported no effect of AHL to the binding activity of SdiA toward the promoter region of *ftsQAZ* operon, and that the AHLs increase the transcriptional activity of SdiA by promoting protein stability rather than by affecting the DNA-binding affinity of the protein. However, it is important to remind that in our study, EMSA was performed using the synthetic AHL *N*-Octanoyl-L-homoserine lactone (C8-HSL). Given the variety of AHL molecules according to the degree of oxidation and saturation and the length of the N-acyl side chains, further studies with other AHLs are needed to explore in more detail whether and how these autoinducers can modulate the DNA-binding activity of SdiA in *K. pneumoniae*.

The production of AI-2 signaling molecules was indirectly measured in the wild-type and mutant *K. pneumoniae* strains by performing bioluminescence assays with the AI-2 reporter strain *Vibrio campbellii*. We observed that when a functional SdiA is present, the production of AI-2 increases as the phases of bacterial growth progress. This increase in AI-2 production is consistent with the role of these signaling molecules as monitors of cell population density (Xavier and Bassler, 2005a; Rutherford and Bassler, 2012; Kendall and Sperandio, 2014). Our results are in accordance with a previous report indicating maximal accumulation of AI-2 by *K. pneumoniae* in the late-exponential phase (Balestrino et al., 2005). Intriguingly, *K. pneumoniae* cells without SdiA regulator show constant production of AI-2 molecules at maximum levels, regardless of the growth phases of the bacteria. Bacteria control population density by sensing and responding to AI-1 and AI-2 QS signaling molecules. The activation of the AI-2 QS system on the mutant strain, as revealed by the maximum production of AI-2 molecules, seems to indicate an attempt by the mutant strain to compensate for the loss of cell density control due to the SdiA absence.

Traditionally, intra- and inter-species communications in Gram-negative bacteria are attributed to QS systems mediated by type 1 and type 2 autoinducers, respectively. How these two QS regulatory systems are connected is controversial and remains poorly understood. While some authors report that *sdiA* and *luxS* work independently (Surette and Bassler, 1999), others suggest that SdiA plays a role in regulating AI-2 uptake and processing (Smith et al., 2010). For instance, DeLisa and coauthors observed that exogenous AI-2 slightly activates the transcription of *sdiA* in an *E. coli luxS* mutant strain (DeLisa et al., 2001), although it cannot be excluded that this activation is due to metabolic changes in the methyl cycle in the *luxS* mutant. More recently, Zhou and colleagues suggested that AI-1 and AI-2 QS systems might be linked in *E. coli* through a synergistic action of SdiA and YdiV to regulate the intracellular concentration cAMP (Zhou et al., 2008). According to these authors, SdiA activates *ydiV* expression by binding on its promoter region in the presence of exogenous AI-1 signaling molecules. YdiV, an EAL domain protein, regulates the production of cAMP that, in turn, positively regulates the expression of *lsrR* and the *lsr* operon. In the present study, we observed up-regulation of *luxS* in the mutant strain throughout the growth stages and slightly induction of *lsrR* at the initial phase of growth. We also observed the DNA-binding activity of SdiA on the *luxS* promoter region and the intergenic region between *lsrR* and *lsr* operon. Although the results presented here seem to indicate a possible direct role of SdiA in the synthesis, uptake, and processing of AI-2 molecules, more studies need to be performed to further strengthen this hypothesis and to better understand how SdiA plays a role in the interaction of AI-1 and AI-2 QS systems in *K. pneumoniae*.

Indeed, the regulatory circuits that integrate and command both AI-1 and AI-2 QS systems are unknown and likely complex. Although LuxR regulators have been originally associated with intra-species signaling, SdiA is a LuxR-type regulator involved not only in inter-species signaling (Michael et al., 2001; Dyszel et al., 2010; Lu et al., 2017) but also in interkingdom communication and according to environmental cues (Smith et al., 2008; Ghosh et al., 2009; Hughes et al., 2010; Wang et al., 2020). SdiA is an orphan LuxR-type regulator encoded by bacteria that do not produce their own AHL and, as such, do not detect endogenous AHL but autoinducers produced by other bacteria. Structural analyses indicate that the ligand-binding domain of SdiA is wide and open enough to accommodate a variety of AHLs molecules, and it can sense and respond to a variety of ligands that can be not only exogenous or synthetic AHL, but also non-AHL chemical compounds (Kim et al., 2013; Nguyen et al., 2015; Styles et al., 2020). This ability to respond to a range of molecules and environmental signals is consistent with the possible role of SdiA as a master modulator for both intra- and inter-species communication.

In summary, *K. pneumoniae* encodes SdiA, an orphan LuxR-type QS regulator since *K. pneumoniae* does not produce its own AHL autoinducers. Nonetheless, SdiA recognizes and responds to AHL produced by other species, indicating some level of inter-species cell-cell communication mediated by SdiA. We herein showed the role of the SdiA regulator in the pathogenesis of *K. pneumoniae* by controlling fimbriae expression, biofilm formation, and production of QS autoinducers. We also determined for the first time the SdiA binding sites within the promoter region of type 1 fimbrial gene cluster *fim*, the *ftsQAZ* cell division gene cluster, and the *luxS* and *lsrA*-*lsrR*, genes related to the synthesis and processing of AI-2 molecules in *K. pneumoniae*. As SdiA detects and responds to AHL produced by other species, we suppose that the modulation of these virulence factors may be orchestrated in a coordinated manner via SdiA-mediated inter-species communication.

## Data Availability Statement

The raw data supporting the conclusions of this article will be made available by the authors, without undue reservation.

## Author Contributions

LFCF and TP conceived and designed the experiments. TP, AEIG, NMGS, and LA executed the experiments and the analysis. MD and HV contributed with reagents, materials, and analysis tools. LFCF and TP wrote the manuscript. MD and HV assisted with critical revision of the manuscript and LFCF coordinated its revision. All authors contributed to the manuscript revision and approved the submitted version.

## Conflict of Interest

The authors declare that the research was conducted in the absence of any commercial or financial relationships that could be construed as a potential conflict of interest.

## Footnotes

1 http://bioinfo.ut.ee/primer3-0.4.0/

## References

[B1] Abed N. Grépinet O. Canepa S. Hurtado-Escobar G. A. Guichard N. Wiedemann A. (2014). Direct regulation of the *pefI*-*srgC* operon encoding the Rck invasin by the quorum-sensing regulator SdiA in *Salmonella Typhimurium*. *Mol. Microbiol.* 94 254–271. 10.1111/mmi.12738 25080967

[B2] Ahmer B. M. M. (2004). Cell-to-cell signalling in *Escherichia coli* and *Salmonella enterica*. *Mol. Microbiol.* 52 933–945. 10.1111/j.1365-2958.2004.04054.x 15130116

[B3] Alcántar-Curiel M. D. Blackburn D. Saldaña Z. Gayosso-Vázquez C. Iovine N. M. De la Cruz M. A. (2013). Multi-functional analysis of *Klebsiella pneumoniae* fimbrial types in adherence and biofilm formation. *Virulence* 4 129–138. 10.4161/viru.22974 23302788 PMC3654611

[B4] Antunes L. C. M. Ferreira R. B. R. Buckner M. M. C. Finlay B. B. (2010). Quorum sensing in bacterial virulence. *Microbiology* 156 2271–2282. 10.1099/mic.0.038794-0 20488878

[B5] Balestrino D. Haagensen J. A. Rich C. Forestier C. (2005). Characterization of type 2 quorum sensing in *Klebsiella pneumoniae* and relationship with biofilm formation. *J. Bacteriol.* 187 2870–2880. 10.1128/JB.187.8.2870-2880.2005 15805533 PMC1070389

[B6] Bassler B. L. Wright M. Showalter R. E. Silverman M. R. (1993). Intercellular signalling in *Vibrio harveyi*: sequence and function of genes regulating expression of luminescence. *Mol. Microbiol.* 9 773–786. 10.1111/j.1365-2958.1993.tb01737.x 8231809

[B7] Culler F. H. Couto C. F. S. Higa S. J. Ruiz M. R. Yang J. M. Bueris V. (2018). Role of SdiA on biofilm formation by atypical enteropathogenic *Escherichia coli*. *Genes* 9:253. 10.3390/genes9050253 29762495 PMC5977193

[B8] De Araujo C. Balestrino D. Roth L. Charbonnel N. Forestier C. (2010). Quorum sensing affects biofilm formation through lipopolysaccharide synthesis in *Klebsiella pneumoniae*. *Res. Microbiol.* 161 595–603. 10.1016/j.resmic.2010.05.014 20600864

[B9] DeLisa M. P. Wu C. F. Wang L. Valdes J. J. Bentley W. E. (2001). DNA microarray-based identification of genes controlled by autoinducer 2-stimulated quorum sensing in *Escherichia coli*. *J. Bacteriol.* 183 5239–5247. 10.1128/jb.183.18.5239-5247.2001 11514505 PMC95404

[B10] Dyszel J. L. Soares J. A. Swearingen M. C. Lindsay A. Smith J. N. Ahmer B. M. (2010). *E. coli* K-12 and EHEC genes regulated by SdiA. *PLoS One* 5:e8946. 10.1371/journal.pone.0008946 20126629 PMC2812512

[B11] Engebrecht J. Silverman M. (1984). Identification of genes and gene products necessary for bacterial bioluminescence. *Proc. Natl. Acad. Sci. U. S. A.* 81 4154–4258. 10.1073/pnas.81.13.4154 6377310 PMC345387

[B12] Fuqua C. (2006). The QscR quorum-sensing regulon of *Pseudomonas aeruginosa*: an orphan claims its identity. *J. Bacteriol.* 188 3169–3171. 10.1128/JB.188.9.3169-3171.2006 16621807 PMC1447470

[B13] Ghosh D. Roy K. Williamson K. E. Srinivasiah S. Wommack K. E. Radosevich M. (2009). Acyl-homoserine lactones can induce virus production in lysogenic bacteria: an alternative paradigm for prophage induction. *Appl. Environ. Microbiol.* 75 7142–7152. 10.1128/AEM.00950-09 19783745 PMC2786502

[B14] Gomes ÉI. Pacheco T. Santos C.d.S.d Pereira J. A. Ribeiro M. L. Darrieux M. (2021). Functional insights from Kpfr, a new transcriptional regulator of fimbrial expression that is crucial for *Klebsiella pneumoniae* pathogenicity. *Front. Microbiol.* 11:601921. 10.3389/fmicb.2020.601921 33552015 PMC7861041

[B15] Gomes A. ÉI. Stuchi L. P. Siqueira N. M. G. Henrique J. B. Vicentini R. Ribeiro M. L. (2018). Selection and validation of reference genes for gene expression studies in *Klebsiella pneumoniae* using Reverse Transcription Quantitative real-time PCR. *Sci. Rep.* 8:9001. 10.1038/s41598-018-27420-2 29899556 PMC5998039

[B16] Guilhen C. Charbonnel N. Parisot N. Gueguen N. Iltis A. Forestier C. (2016). Transcriptional profiling of *Klebsiella pneumoniae* defines signatures for planktonic, sessile and biofilm-dispersed cells. *BMC Genom.* 17:237. 10.1186/s12864-016-2557-x 26979871 PMC4791964

[B17] Hall-Stoodley L. Costerton J. W. Stoodley P. (2004). Bacterial biofilms: from the natural environment to infectious diseases. *Nat. Rev. Microbiol.* 2 95–108. 10.1038/nrmicro821 15040259

[B18] Holt K. E. Wertheim H. Zadoks R. N. Baker S. Whitehouse C. A. Dance D. (2015). Genomic analysis of diversity, population structure, virulence, and antimicrobial resistance in *Klebsiella pneumoniae*, an urgent threat to public health. *Proc. Natl. Acad. Sci. U. S. A.* 112 E3574–E3581. 10.1073/pnas.1501049112 26100894 PMC4500264

[B19] Hughes D. T. Terekhova D. A. Liou L. Hovde C. J. Sahl J. W. Patankar A. V. (2010). Chemical sensing in mammalian host-bacterial commensal associations. *Proc. Natl. Acad. Sci. U. S. A.* 107 9831–9836. 10.1073/pnas.1002551107 20457895 PMC2906910

[B20] Janssens J. C. A. Metzger K. Daniels R. Ptacek D. Verhoeven T. Habel L. W. (2007). Synthesis of N-acyl homoserine lactone analogues reveals strong activators of SdiA, the *Salmonella enterica* serovar *Typhimurium* LuxR homologue. *Appl. Environ. Microbiol.* 73:535. 10.1128/AEM.01451-06 17085703 PMC1796990

[B21] Joseleau-Petit D. Vinella D. D’Ari R. (1999). Metabolic alarms and cell division in *Escherichia coli*. *J. Bacteriol.* 181 9–14. 10.1128/JB.181.1.9-14.1999 9864306 PMC103525

[B22] Kanamaru K. Tatsuno I. Tobe T. Sasakawa C. (2000). SdiA, an *Escherichia coli* homologue of quorum-sensing regulators, controls the expression of virulence factors in enterohaemorrhagic *Escherichia coli* O157:H7. *Mol. Microbiol.* 38 805–816. 10.1046/j.1365-2958.2000.02171.x 11115115

[B23] Kendall M. Sperandio V. (2014). Cell-to-cell signaling in *Escherichia coli* and *Salmonella*. *EcoSal Plus* 6 1–15. 10.1128/ecosalplus.ESP-0002-2013 26442936 PMC4229655

[B24] Keynan Y. Rubinstein E. (2007). The changing face of *Klebsiella pneumoniae* infections in the community. *Int. J. Antimicrob. Agents* 30 385–389. 10.1016/j.ijantimicag.2007.06.019 17716872

[B25] Kim T. Duong T. Wu C. A. Choi J. Lan N. Kang S. W. (2013). Structural insights into the molecular mechanism of *Escherichia coli* SdiA, a quorum-sensing receptor Acta Crystallogr. *D Biol. Crystallogr.* 70 694–707. 10.1107/S1399004713032355 24598739

[B26] Kohli N. Crisp Z. Riordan R. Li M. Alaniz R. C. Jayaraman A. (2018). The microbiota metabolite indole inhibits *Salmonella* virulence: involvement of the PhoPQ two-component system. *PLoS One* 13:e0190613. 10.1371/journal.pone.0190613 29342189 PMC5771565

[B27] Lee J.-H. Lequette Y. Greenberg E. P. (2006). Activity of purified QscR, a *Pseudomonas aeruginosa* orphan quorum-sensing transcription factor. *Mol. Microbiol.* 59 602–609. 10.1111/j.1365-2958.2005.04960.x 16390453

[B28] Lee J. Jayaraman A. Wood T. K. (2007). Indole is an inter-species biofilm signal mediated by SdiA. *BMC Microbiol.* 7:42. 10.1186/1471-2180-7-42 17511876 PMC1899176

[B29] Lee J. Maeda T. Hong S. H. Wood T. (2009). Reconfiguring the quorum-sensing regulator SdiA of *Escherichia coli* to control biofilm formation via indole and N-acylhomoserine lactones. *Appl. Environ. Microbiol.* 75 1703–1716. 10.1128/AEM.02081-08 19168658 PMC2655446

[B30] Li B. Zhao Y. Liu C. Chen Z. Zhou D. (2014). Molecular pathogenesis of *Klebsiella pneumoniae*. *Fut. Microbiol.* 9 1071–1081. 10.2217/fmb.14.48 25340836

[B31] Lin W. H. Wang M. C. Tseng C. C. Ko W. C. Wu A. B. Zheng P. X. (2010). Clinical and microbiological characteristics of *Klebsiella pneumoniae* isolates causing community-acquired urinary tract infections. *Infection* 38 459–464. 10.1007/s15010-010-0049-5 20734217

[B32] Livak K. J. Schmittgen T. D. (2001). Analysis of relative gene expression data using real-time quantitative PCR and the 2(-Delta Delta C(T)) method. *Methods* 25 402–408. 10.1006/meth.2001.1262 11846609

[B33] Lu Y. Zeng J. Wu B. E. S. Wang L. Cai R. (2017). Quorum sensing N-acyl homoserine lactones-SdiA suppresses *Escherichia coli-Pseudomonas aeruginosa* conjugation through inhibiting *traI* expression. *Front. Cell. Infect. Microbiol.* 7:7. 10.3389/fcimb.2017.00007 28164039 PMC5247672

[B34] Ma X. Zhang S. Xu Z. Li H. Xiao Q. Qiu F. (2020). SdiA improves the acid tolerance of *E. coli* by regulating GadW and GadY expression. *Front. Microbiol.* 11:1078. 10.3389/fmicb.2020.01078 32582066 PMC7286202

[B35] Michael B. Smith J. N. Swift S. Heffron F. Ahmer B. M. (2001). SdiA of *Salmonella enterica* is a LuxR homolog that detects mixed microbial communities. *J. Bacteriol.* 183 5733–5742. 10.1128/jb.183.19.5733-5742.2001 11544237 PMC95466

[B36] Miller M. B. Bassler B. L. (2001). Quorum Sensing in Bacteria. *Annu. Rev. Microbiol.* 55 165–199. 10.1146/annurev.micro.55.1.165 11544353

[B37] Ng W.-L. Bassler B. L. (2009). Bacterial quorum-sensing network architectures. *Annu. Rev. Genet.* 43 197–222. 10.1146/annurev-genet-102108-134304 19686078 PMC4313539

[B38] Nguyen Y. Nguyen N. X. Rogers J. L. Liao J. MacMillan J. B. Jiang Y. (2015). Structural and mechanistic roles of novel chemical ligands on the Sdia quorum-sensing transcription regulator. *mBio* 6 :e02429–14. 10.1128/mBio.02429-14 25827420 PMC4453555

[B39] Paczosa M. K. Mecsas J. (2016). *Klebsiella pneumoniae*: going on the offense with a strong defense. *Microbiol. Mol. Biol. Rev.* 80 629–661. 10.1128/MMBR.00078-15 27307579 PMC4981674

[B40] Patankar A. V. González J. E. (2009). Orphan LuxR regulators of quorum sensing. *FEMS Microbiol. Rev.* 33 739–756. 10.1111/j.1574-6976.2009.00163.x 19222586

[B41] Pereira C. S. de Regt A. K. Brito P. H. Miller S. T. Xavier K. B. (2009). Identification of functional LsrB-like autoinducer-2 receptors. *J. Bacteriol.* 191 6975–6987. 10.1128/JB.00976-09 19749048 PMC2772480

[B42] Pereira C. S. Thompson J. A. Xavier K. B. (2013). AI-2-mediated signalling in bacteria. *FEMS Microbiol. Rev.* 37 156–181. 10.1111/j.1574-6976.2012.00345.x 22712853

[B43] Rosen D. A. Pinkner J. S. Jones J. M. Walker J. N. Clegg S. Hultgren S. J. (2008). Utilization of an intracellular bacterial community pathway in *Klebsiella pneumoniae* urinary tract infection and the effects of FimK on type 1 pilus expression. *Infect. Immun.* 76 3337–3345. 10.1128/IAI.00090-08 18411285 PMC2446714

[B44] Rozen S. Skaletsky H. (2000). Primer3 on the WWW for general users and for biologist programmers. *Methods Mol. Biol.* 132 365–386. 10547847 10.1385/1-59259-192-2:365

[B45] Rutherford S. T. Bassler B. L. (2012). Bacterial quorum sensing: its role in virulence and possibilities for its control. *Cold Spring Harb. Perspect. Med.* 2:a012427. 10.1101/cshperspect.a012427 23125205 PMC3543102

[B46] Sabag-Daigle A. Ahmer B. M. M. (2012). ExpI and PhzI are descendants of the long lost cognate signal synthase for SdiA. *PLoS One* 7:e47720. 10.1371/journal.pone.0047720 23082201 PMC3474713

[B47] Sabag-Daigle A. Soares J. A. Smith J. N. Elmasry M. E. Ahmer B. M. M. (2012). The acyl homoserine lactone receptor. *SdiA, of Escherichia coli* and *Salmonella enterica* Serovar Typhimurium does not respond to indole. *Appl. Environ. Microbiol.* 78 5424–5431. 10.1128/AEM.00046-12 22610437 PMC3416396

[B48] Sabag-Daigle A. Dyszel J. L. Gonzalez J. F. Ali M. M. Ahmer B. M. M. (2015). Identification of sdiA-regulated genes in a mouse commensal strain of *Enterobacter cloacae*. *Front. Cell. Infect. Microbiol.* 5:47. 10.3389/fcimb.2015.00047 26075189 PMC4444967

[B49] Schauder S. Shokat K. Surette M. G. Bassler B. L. (2001). The LuxS family of bacterial autoinducers: biosynthesis of a novel quorum-sensing signal molecule. *Mol. Microbiol.* 41 463–476. 10.1046/j.1365-2958.2001.02532.x 11489131

[B50] Schroll C. Barken K. B. Krogfelt K. A. Struve C. (2010). Role of type 1 and type 3 fimbriae in *Klebsiella pneumoniae* biofilm formation. *BMC Microbiol.* 10:179. 10.1186/1471-2180-10-179 20573190 PMC2911432

[B51] Schuster M. Urbanowski M. L. Greenberg E. P. (2004). Promoter specificity in *Pseudomonas aeruginosa* quorum sensing revealed by DNA binding of purified LasR. *Proc. Natl. Acad. Sci. U. S. A.* 101 15833–15839. 15505212 10.1073/pnas.0407229101 PMC528741

[B52] Sharma V. K. Bearson S. M. D. Bearson B. L. (2010). Evaluation of the effects of *sdiA*, a luxR homologue, on adherence and motility of *Escherichia coli* O157:H7. *Microbiology* 156 1303–1312. 10.1099/mic.0.034330-0 20110300

[B53] Shimada T. Shimada K. Matsui M. Kitai Y. Igarashi J. Suga H. (2014). Roles of cell division control factor SdiA: recognition of quorum sensing signals and modulation of transcription regulation targets. *Genes Cells* 19 405–418. 10.1111/gtc.12139 24645791

[B54] Sitnikov D. M. Schineller J. B. Baldwin T. O. (1996). Control of cell division in *Escherichia coli*: regulation of transcription of *ftsQA* involves both *rpoS* and SdiA-mediated autoinduction. *Proc. Natl. Acad. Sci. U.S.A.* 93 336–341. 10.1073/pnas.93.1.336 8552633 PMC40233

[B55] Smith J. N. Ahmer B. M. M. (2003). Detection of other microbial species by *Salmonella*: expression of the SdiA regulon. *J. Bacteriol.* 185 1357–1366. 12562806 10.1128/JB.185.4.1357-1366.2003 PMC142872

[B56] Smith J. N. Dyszel J. L. Soares J. A. Ellermeier C. D. Altier C. Lawhon S. D. (2008). SdiA, an N-acylhomoserine lactone receptor, becomes active during the transit of *Salmonella enterica* through the gastrointestinal tract of turtles. *PLoS One* 3:e2826. 10.1371/journal.pone.0002826 18665275 PMC2475663

[B57] Smith J. L. Fratamico P. M. Yan X. (2010). Eavesdropping by bacteria: the role of SdiA in *Escherichia coli* and *Salmonella enterica* serovar Typhimurium quorum sensing. *Foodborne Pathog. Dis.* 8 169–178. 10.1089/fpd.2010.0651 21034261

[B58] Stahlhut S. G. Struve C. Krogfelt K. A. Reisner A. (2012). Biofilm formation of *Klebsiella pneumoniae* on urethral catheters requires either type 1 or type 3 fimbriae. *FEMS Immunol. Med. Microbiol.* 65 350–359. 10.1111/j.1574-695X.2012.00965.x 22448614 PMC3410544

[B59] Struve C. Bojer M. Krogfelt K. A. (2008). Characterization of *Klebsiella pneumoniae* type 1 fimbriae by detection of phase variation during colonization and infection and impact on virulence. *Infect. Immun.* 76 4055–4065. 10.1128/IAI.00494-08 18559432 PMC2519443

[B60] Struve C. Bojer M. Krogfelt K. A. (2009). Identification of a conserved chromosomal region encoding *Klebsiella pneumoniae* type 1 and type 3 fimbriae and assessment of the role of fimbriae in pathogenicity. *Infect. Immun.* 77 5016–5024. 10.1128/IAI.00585-09 19703972 PMC2772557

[B61] Styles M. J. Early S. A. Tucholski T. West K. H. J. Ge Y. Blackwell H. E. (2020). Chemical control of quorum sensing in *E. coli*: identification of small molecule modulators of Sdia and mechanistic characterization of a covalent inhibitor. *ACS Infect. Dis.* 6 3092–3103. 10.1021/acsinfecdis.0c00654 33124430 PMC7736514

[B62] Surette M. G. Bassler B. L. (1999). Regulation of autoinducer production in *Salmonella typhimurium*. *Mol. Microbiol.* 31 585–595. 10.1046/j.1365-2958.1999.01199.x 10027975

[B63] Tavio M. M. Aquili V. D. Poveda J. B. Antunes N. T. Sanchez-Cespedes J. Vila J. (2010). Quorum-sensing regulator *sdiA* and *marA* overexpression is involved in in vitro-selected multidrug resistance of *Escherichia coli*. *J. Antimicrob. Chemother.* 65 1178–1186. 10.1093/jac/dkq112 20395215

[B64] Urbanowski M. L. Lostroh C. P. Greenberg E. P. (2004). Reversible acyl-homoserine lactone binding to purified *Vibrio fischeri* LuxR protein. *J. Bacteriol.* 186 631–637. 14729687 10.1128/JB.186.3.631-637.2004 PMC321501

[B65] van der Woude M. W. Baumler A. J. (2004). Phase and antigenic variation in bacteria. *Clin. Microbiol. Rev.* 17 581–611. 10.1128/CMR.17.3.581-611.2004 15258095 PMC452554

[B66] Wang X. D. de Boer P. A. Rothfield L. I. (1991). A factor that positively regulates cell division by activating transcription of the major cluster of essential cell division genes of *Escherichia coli*. *Embo J.* 10 3363–3372. 1915297 10.1002/j.1460-2075.1991.tb04900.x PMC453064

[B67] Wang L. Hashimoto Y. Tsao C.-Y. Valdes J. J. Bentley W. E. (2005). Cyclic AMP (cAMP) and cAMP receptor protein influence both synthesis and uptake of extracellular autoinducer 2 in *Escherichia coli*. *J. Bacteriol.* 187 2066–2076. 10.1128/JB.187.6.2066-2076.2005 15743955 PMC1064054

[B68] Wang S. Payne G. F. Bentley W. E. (2020). Quorum sensing communication: molecularly connecting cells, their neighbors, and even devices. *Annu. Rev. Chem. Biomol. Eng.* 11 447–468. 10.1146/annurev-chembioeng-101519-124728 32168999

[B69] Wu C. Lokanath N. K. Kim D. Y. Nguyen L. D. N. Kim K. K. (2008). Crystallization and preliminary X-ray studies of SdiA from *Escherichia coli* Acta Crystallogr. *Sect. F Struct. Biol. Cryst. Commun.* 64 19–21. 10.1107/S1744309107059696 18097094 PMC2373988

[B70] Xavier K. B. Bassler B. L. (2005b). Regulation of uptake and processing of the quorum-sensing autoinducer AI-2 in *Escherichia coli*. *J. Bacteriol.* 187 238–248. 10.1128/JB.187.1.238-248.2005 15601708 PMC538819

[B71] Xavier K. B. Bassler B. L. (2005a). Interference with AI-2-mediated bacterial cell-cell communication. *Nature* 437 750–753. 10.1038/nature03960 16193054 PMC1388276

[B72] Yamamoto K. Yata K. Fujita N. Ishihama A. (2001). Novel mode of transcription regulation by SdiA, an *Escherichia coli* homologue of the quorum-sensing regulator. *Mol. Microbiol.* 41 1187–1198. 10.1046/j.1365-2958.2001.02585.x 11555297

[B73] Yao Y. Martinez-Yamout M. A. Dickerson T. J. Brogan A. P. Wright P. E. Dyson H. J. (2006). Structure of the *Escherichia coli* quorum sensing protein SdiA: activation of the folding switch by acyl homoserine lactones. *J. Mol. Biol.* 355 262–273. 10.1016/j.jmb.2005.10.041 16307757

[B74] Yao Y. Dickerson T. J. Hixon M. S. Dyson H. J. (2007). NMR detection of adventitious xylose binding to the quorum-sensing protein SdiA of *Escherichia coli*. *Bioorg. Med. Chem. Lett.* 17 6202–6205. 10.1016/j.bmcl.2007.09.029 17889538 PMC2249169

[B75] Zhou X. Meng X. Sun B. (2008). An EAL domain protein and cyclic AMP contribute to the interaction between the two quorum sensing systems in *Escherichia coli*. *Cell Res.* 18 937–948. 10.1038/cr.2008.67 18560382

[B76] Zhu J. Winans S. C. (2001). The quorum-sensing transcriptional regulator TraR requires its cognate signaling ligand for protein folding, protease resistance, and dimerization. *Proc. Natl. Acad. Sci. U. S. A.* 98 1507–1512. 10.1073/pnas.98.4.1507 11171981 PMC29287

[B77] Zhu H. Shen Y. L. Wei D. Z. Zhu J. W. (2008). Inhibition of quorum sensing in *Serratia marcescens* H30 by molecular regulation. *Curr. Microbiol.* 56 645–650. 10.1007/s00284-008-9140-x 18320272

[B78] Zhu H. Liu H. J. Ning S. J. Gao Y. L. (2012). The response of type 2 quorum sensing in *Klebsiella pneumoniae* to a fluctuating culture environment. *DNA Cell Biol.* 31 455–459. 10.1089/dna.2011.1375 21877918

