# Supplementary material for: SdiA, a Quorum-Sensing Regulator, Suppresses Fimbriae Expression, Biofilm Formation, and Quorum-Sensing Signaling Molecules Production in Klebsiella pneumoniae
Source: Front Microbiol. 2021 Jun 21;12:597735. doi: 10.3389/fmicb.2021.597735 (PMC8255378; doi:10.3389/fmicb.2021.597735)
Supplement: Supplementary file 1 [file Table_1.docx]

**Supplementary Table S1.** The most efficient insertion site of the re-targeted intron on *sdiA*, as ranked by TargeTron Design website.

| **Position ^1^** | **Target sequence** | **Score** | **E-value** |
| --- | --- | --- | --- |
| 185-186 | CCGCGCATGGATGTCGCACTATCAGGCAGA-***intron***-AAATTATTTCGCGAT | 7.55 | 0.152 |

1. Nucleotide position of the RNA intron insertion at the sense strand of *sdiA* gene.
