# Supplementary material for: SdiA, a Quorum-Sensing Regulator, Suppresses Fimbriae Expression, Biofilm Formation, and Quorum-Sensing Signaling Molecules Production in Klebsiella pneumoniae
Source: Front Microbiol. 2021 Jun 21;12:597735. doi: 10.3389/fmicb.2021.597735 (PMC8255378; doi:10.3389/fmicb.2021.597735)
Supplement: Supplementary file 2 [file Table_2.docx]

**Supplementary Table S2.** Nucleotide sequence of the primers generated by TargeTron Design website and used in PCR reactions to mutate (re-target) the intron.

| ***Primers*** | **Nucleotide sequences (5’>3’)** |
| --- | --- |
| IBS-*sdiA* | AAAAAAGCTTATAATTATCCTTACTATCCGGCAGAGTGCGCCCAGATAGGGTG |
| EBS1d-*sdiA* | TGAACGCAAGTTTCTAATTTCGGTTGATAGTCGATAGAGGAAAGTGTCT |
| EBS2-*sdiA* | CAGATTGTACAAATGTGGTGATAACAGATAAGTCGGCCAGAAATAACTTACCTTTCTTTGT |
| EBS Universal | CGAAATTAGAAACTTGCGTTCAGTAAAC |
