# Supplementary material for: SdiA, a Quorum-Sensing Regulator, Suppresses Fimbriae Expression, Biofilm Formation, and Quorum-Sensing Signaling Molecules Production in Klebsiella pneumoniae
Source: Front Microbiol. 2021 Jun 21;12:597735. doi: 10.3389/fmicb.2021.597735 (PMC8255378; doi:10.3389/fmicb.2021.597735)
Supplement: Supplementary file 3 [file Table_3.doc]

**Supplementary Table S3.** Primers used on this study.

| **Purpose** | **Primers** | **Sequence 5’-3’** | **Tm**  **(^o^C)** | **Size (bp)** |
| --- | --- | --- | --- | --- |
| Amplification of the *sdiA* coding region plus 3’ and 5’ flanking regions to generate the complemented strain *sdiA*::*kan*^R^_comp_ | *sdiA*comp-F | GTATACACCGCTTCCCATCG | 58 | 994 |
|  | *sdiA*comp-R | CTGTGCCGGCTGAAAGTATC |  |  |
|  |  |  |  |  |
| Amplification of the fimS invertible element from the *fim* gene cluster for the phase variation assay **^1^** | CAS168 | GGGACAGATACGCGTTTGAT | 55 | 817 |
|  | CAS169 | GGCCTAACTGAACGGTTTGA |  |  |
| RT-qPCR reactions **^2^** | *sdiA*-F | TAAGCTTGCTGACGCTGCT | 60 | 97 |
|  | *sdiA*-R | AAATCCTCAAATGGACGGC |  |  |
|  | *luxS*-F | TTCTATATGAGCCTGATTGGTACG | 60 | 101 |
|  | *luxS*-R | ATCTGGTTCTGATCTTTCACCTTC |  |  |
|  | *fimA*-F | CGTCGGTTTCAACATCCAG | 60 | 100 |
|  | *fimA*-R | GGTGGTATTGCTGCTGTCG |  |  |
|  | *ecpA*-F | AATATTATGGGCGGCAACCT | 60 | 98 |
|  | *ecpA*-R | CCGCTGATGATGGAGAAAGT |  |  |
|  | *mrkA*-F | GGCTGCTGATGGCACTAAA | 60 | 105 |
|  | *mrkA*-R | GCCAGGTAGCCCTGTTGTT |  |  |
|  | *ftsQ*-F | AGCAGGGCTATTGGAAGGAT | 60 | 109 |
|  | *ftsQ*-R | TCAGCGCTAAGTTGACTGAATG |  |  |
|  | *rpoS*-F | ATGATTTAAATGAAGACGCGGA | 60 | 110 |
|  | *rpoS*-R | CAGCTCTTCCTCAGCCAGAT |  |  |
|  | *rho*-F | AACTACGACAAGCCGGAAAA | 60 | 99 |
|  | *rho*-R | ACCGTTACCACGCTCCATAC |  |  |
|  | *lsrB*-F | GGGCGTGGATGTCACCTAT | 60 | 115 |
|  | *lsrB*-R | GAGACGGCGGAAACGATAAT |  |  |
|  | *lsrR*-F | TGACGCAAAATGACATCGGC | 60 | 118 |
|  | *lsrR*-R | CCCTCATAGCGCGAGTTGAT |  |  |
|  | *luxS*-F | TTCTATATGAGCCTGATTGGTACG | 60 | 101 |
|  | *luxS*-R | ATCTGGTTCTGATCTTTCACCTTC |  |  |
|  |  |  |  |  |
| Amplification of the *sdiA* coding region for expression and purifica-tion of the SdiA protein **^3^** | exSdiA-F | AGCCATATGCTGCACCAATTTCA | 66 | 703 |
|  | exSdiA-R | TATTCTCGAGTCAGATTAGCCCAGTCG |  |  |
| Amplification of the promoter region of the selected genes and the negative control **^4^** for EMSA | *sdiA*prom-F | GTATACACCGCTTCCCATCG | 58 | 308 |
|  | *sdiA*prom-R | GGTTTCACGTTGCAGGAGAT |  |  |
|  | *luxS*prom-F | AAGAGCCGCTGGAGATCC | 58 | 330 |
|  | *luxS*prom-R | CTTCCATTCGGGTATGGTCG |  |  |
|  | *fimA*prom-F | GAAAACTGTGCAGAGCCGG | 58 | 200 |
|  | *fimA*prom-R | GTTGACCGTGGTGGTATCG |  |  |
|  | *ftsQ*prom-F | TAATCCCAAGAGTGTGACATGG | 58 | 296 |
|  | *ftsQ*prom-R | TCAGCGCTAAGTTGACTGAATG |  |  |
|  | *lsrAR*prom-F | ATGATGAAGCCACTGCTTGAA | 58 | 339 |
|  | *lsrAR*prom-R | AGCGGTTCTCTTCCGTAATG |  |  |
|  | M13-F | GTTTTCCCAGTCACGAC | 55 | 220 |
|  | M13-R | CAGGAAACAGCTATGAC |  |  |
|  |  |  |  |  |

1. The CAS primers were based on Struve, Bojer & Krogfelt (2008, doi: 10.1128/IAI.00494-08).
2. Primers for RT-qPCR analysis were designed using *Primer3 version 4.1.0* web-program (available at http://bioinfo.ut.ee/primer3-0.4.0/; ROZEN & SKALETSKY, 2000, doi: 10.1385/1-59259-192-2:365), in order to present 60 ^o^C of annealing temperature and amplicon sizes ranging from 95 to 120 base pairs (bp).
3. The underlined nucleotides indicate the restriction sites for *Nde*I (forward primer) and *Xho*I (reverse primer).
4. The 220 bp amplicon of the negative control was obtained by PCR amplifying a recircularized pCR^TM^2.1 vector using primers M13.
