# Supplementary material for: SdiA, a Quorum-Sensing Regulator, Suppresses Fimbriae Expression, Biofilm Formation, and Quorum-Sensing Signaling Molecules Production in Klebsiella pneumoniae
Source: Front Microbiol. 2021 Jun 21;12:597735. doi: 10.3389/fmicb.2021.597735 (PMC8255378; doi:10.3389/fmicb.2021.597735)
Supplement: Supplementary file 4 [file Table_4.docx]

**Supplementary Table S4.** Putatives SdiA-boxes on the promoter region of the indicated genes. These DNA binding sites were identified within the DNA fragments used in EMSA.

| **Genes** | **Sequence of the SdiA-box ^1^** | **Upstream distance from the ATG (bp)** |
| --- | --- | --- |
| ***ftsQ*** | **AAAAT**aaacacgatga**TAACA**  **AAAAG**cagaaacggctc**GTAAA**  **TAAAC**aatcatt**CACAA** | -125  -59  -41 |
| ***luxS*** | **GAAAG**tggaagct**GAAGA**  **AAACT**ttcatctctgattcagggatgatgatgataa**TAAAT** | -214  -122 |
| ***lsrR*** | **AAAAG**cgtagacggtgaactatatttacaaccgtgc**GAAGA**  **AAAAT**gggccagcga**TGAAA** | -172  -101 |
| ***fimA*** | **CAAAG**gaaaacagtAT**GAAAA**  **AAAAC**agtATGAAAAT**CAAAA** | -14  -8 |

1. The putatives SdiA-boxes are underlined and in bold, according to the consensus sequence 5’-AAAA(N)_5-30_AAAA-3’. Nucleotides differing from the AAAA sequence are indicated in red. The spacer nucleotides are shown in lowercase. The start codon of *fimA* is double underlined.
